# Disparate power transmission performance reinforces Italian social inequities

**DOI:** 10.1016/j.isci.2025.112953

**Published:** 2025-06-20

**Authors:** Katherine Emma Lonergan, Andrej Stankovski, Blazhe Gjorgiev, Giovanni Sansavini

**Affiliations:** 1 Reliability and Risk Engineering Laboratory, Institute of Energy and Process Engineering, Department of Mechanical and Process Engineering, ETH Zurich, 8092 Zurich, Switzerland

**Keywords:** energy sustainability, energy management, Energy Modeling

## Abstract

High-voltage electric power transmission systems facilitate the delivery of large shares of electricity to customers and, as such, are key to enabling equitable power service. Here, we investigate the historical performance of the Italian high-voltage power transmission grid delivering power to utility consumers. Using open socio-economic and power system data, we find that socially vulnerable regions experience higher frequency and energy loss than less vulnerable regions. We find that performance disparities stem from infrastructure and terrain characteristics. Components operating at 132 kV and 150 kV levels are more failure-prone than those at other voltage levels, possibly due to high congestion near large cities. Worst-performing substations demonstrate higher rates of failure among non-transmission system operator (TSO)-owned components and due to weather-induced line failures, emphasizing the need for standardized operations and maintenance procedures. Altogether, our work offers practical recommendations for improving energy equity and highlights the critical role of high-voltage transmission in achieving that aim.

## Highlights

• Functioning high-voltage electric power systems support local electricity service
• Vulnerable Italian regions experience worse power system performance
• Performance discrepancies are due to environmental and technical factors

## Graphical abstract

**Figure:**
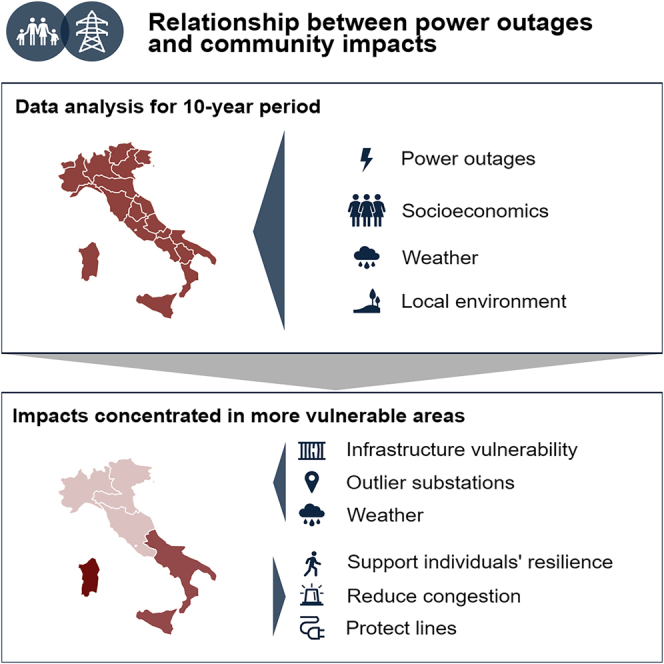

## Introduction

Energy systems enable human well-being by providing basic services (e.g., heating and lighting), as well as supporting other critical infrastructure (e.g., water supply, telecommunications).^1^ At the same time, the unequal provision of these services and the differential impact of their absence can create and reinforce societal inequities.

Achieving *energy equity* or improving community well-being and vulnerability reduction through the provision of power services,^2^ is a worldwide challenge. Ensuring basic electricity access is a persistent key challenge^3^; however, advanced economies also struggle to achieve equitable power service. Major challenges include energy affordability,^4^ increased frequency of disconnections,^5^^,^^6^ and inequitable post-disaster recovery times.^7^^,^^8^^,^^9^^,^^10^^,^^11^ The literature shows that many of these challenges fall on communities that are already considered socially vulnerable, for instance, due to age, health, gender, family background (e.g., migration history and ethnicity), financial resources, housing status, and knowledge of the local language.^12^ These factors are often interrelated and, sometimes, compounding.^4^ Problematically, the factors that make these communities vulnerable in a general sense can also influence their ability to mitigate the occurrence and impact of power failures compared to other populations.^5^^,^^7^

Given the interrelationship between individual characteristics and vulnerability, most energy equity studies consider power system service on the local scale or provided by low-voltage distribution system operators. Adopting this local focus directly supports the identification of energy inequities based on empirical data and facilitates specific recommendations for improvements,^13^ for example, for local electricity system operators and their regulators.^14^^,^^15^^,^^16^

However, achieving equitable power service must extend beyond local systems to consider the performance of the high-voltage transmission system. While transmission systems are comparatively reliable compared to distribution grids,^17^ investigating the high-voltage system through an equity lens is relevant for at least three reasons. First, equitable service at the high-voltage transmission system is necessary but insufficient to ensure equitable power delivery for individuals. There are many opportunities to create consumer inequities at the local level (e.g., though unfair pricing mechanisms^13^), but achieving equitable power service for consumers is much more difficult if the transmission backbone of the electricity system is already operating inequitably. Second, changes made to transmission system performance can disproportionately impact end-users compared to actions taken within the distribution system. Namely, mitigating failures in the high-voltage grid has a larger impact than mitigating failures in lower-voltage systems in terms of both electricity delivery and spatially impacted area. Third, disruption in the high-voltage grid can cause widespread and long-term social and economic damage.^18^ Power outages cause immediate financial losses and impact the availability of other critical infrastructure and facilities like hospitals, water supply, and lighting; however, extended and frequent outages can also halt industrial operations and divert investments to areas with more stable power supply.^19^ Despite the connection between a well-functioning transmission system and equity, the systems have been largely overlooked in energy equity studies.

Here, we take the first steps to close this research gap. To do so, we assess the historical performance of the Italian high-voltage transmission system through an equity lens. Italy offers a strong first case study given the country’s extensive available socioeconomic and power systems data^20^^,^^21^ (see key resources table), and long-standing regional inequalities.^22^ We rely on a dataset of 11,831 failures recorded between 2013 and 2022^14^ affecting electricity utility customers (i.e., excluding failures that affected power stations, industrial sites, and military bases). Focusing on this subset of outages allows us to ask the following research questions.

(1) How have failures in the Italian transmission grid affected utility consumers?
(2) What are the driving causes of failure leading to power loss for utility consumers?
(3) What opportunities exist for correcting existing disparities?

Our results show that utility consumers experienced a value of lost load worth approximately 2.4 €/person/year between 2012 and 2022 due to transmission system failures. Although the absolute inconvenience in frequency and total lost load per consumer is minor, regional differences are significant, with Southern Italy generally experiencing the worst power service. This result is particularly undesirable given that Southern Italy also has a comparatively vulnerable population. Results also show that the worst outages and worst-performing substations cause a disproportionate share of lost energy and are critical weaknesses to be addressed in seeking equitable power service. Strengthening outage-response competencies in organizations owning and managing high-voltage electricity equipment would also support more equal performance.

## Results

### Power system performance assessment

**Figure 1:**
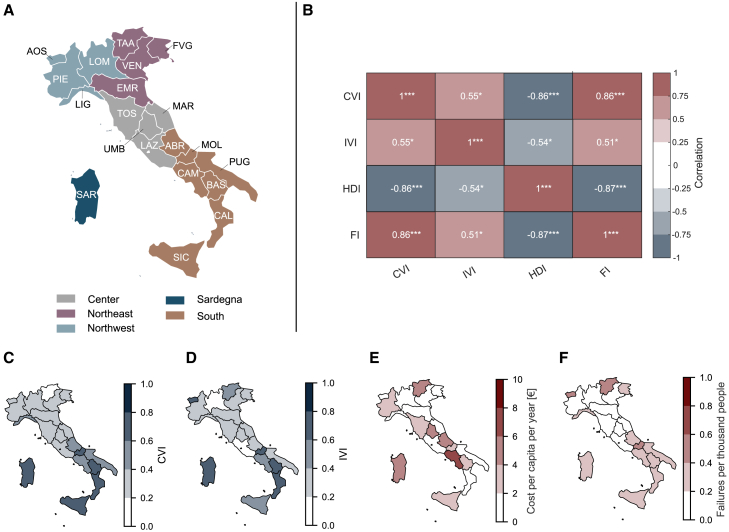
Italian regions, power system performance, and regional vulnerability (A) Italian regions and area groupings. Regions are listed by abbreviation with the full naming key in Table S1. (B) The correlation between the community vulnerability index (CVI), the infrastructure vulnerability index (IVI), the Human Development Index (HDI), and the Fragility Index (FI). ∗*p* value < 0.05, ∗∗*p*-value < 0.01, and ∗∗∗*p*-value < 0.001. The HDI shows a negative correlation with all other indices because it measures human wellbeing, whereas the other three indices measure vulnerability. See characterizing community and infrastructure vulnerability in method details for index calculation. (C and D) The regional CVI and IVI. (E and F) Power system performance according to value of lost load per year and failures per thousand people. See also Figures S1, S3, and S4; Tables S1–S4.

We assess the historical performance of the Italian high-voltage power system in providing electricity for individuals. To do so, our analysis considers failures that impact utility-connected consumers, i.e., as opposed to industrial, military, or government consumers. Failures refer to an unplanned loss of component function due to physical damage or actuation of the protective devices (see Table 1). We consider failures that resulted in demand not served (DNS) and near misses that did not disrupt the supply. Utility-connected consumers include residential consumers and the tertiary sector, like restaurants and schools. As such, our results likely overestimate the impact of failure on individuals alone; they nonetheless represent the best estimate in the absence of more disaggregated data (see limitations of the study). We consider all failures recorded by the Italian transmission system operator (TSO) over a ten-year period spanning 2013–2022, which allows us to understand the trends in transmission system performance. The 11,831 failures are recorded in the high-voltage system, which spans the voltage range of 70 kV–380 kV (see power system failure data in method details). We characterize power system performance and regional vulnerability for Italian regions and areas (Figures 1A and S1; Table S1). To characterize regional vulnerability, we introduce two vulnerability indices. First, we develop the community vulnerability index (CVI) to represent how power outage impacts are experienced by the population. This composite index comprises socio-cultural factors, including poverty rate, average income per inhabitant, access to essential services, population dependency, the share of a healthy population, and the share of the population with low education. Second, we develop the infrastructure vulnerability index (IVI) to represent the factors that could contribute to increased failure rates due to infrastructural challenges. This composite index includes regional gross domestic product (GDP), connection density, vegetation coverage, as well as mountainous and remoteness indicators. Critically, we develop both the CVI and the IVI to specifically describe vulnerability to power outages, e.g., as opposed to indices that describe general social vulnerability^23^^,^^24^ or vulnerability to other infrastructure hazards^25^ (see characterizing community and infrastructure vulnerability in method details and Table S2).

**Table 1:** Power system performance metrics

Outage metric	Definition	Unit
Energy not served	Magnitude of the failure	Megawatt-hours (MWh)
Failure frequency per capita	How often failures occur normalized by population	Failures/year/person

The CVI and IVI show moderate correlation with one another and high absolute correlation with other indices of human vulnerability and wellbeing (Figure 1B). In particular, the CVI exhibits high absolute correlations of over 0.8 with both the Human Development Index (HDI) and the Fragility Index (FI). This high correlation reflects that the CVI, HDI, and FI all aim to describe human wellbeing. The IVI has a comparatively lower correlation with all other indicators, showing that the IVI measures something distinct from the more general vulnerability and wellbeing indicators. These results are driven by the different constituent components of the CVI and the IVI (Figure S2). Nonetheless, regional CVI and IVI indicators show vulnerability to be generally higher in the South of Italy, including the regions of Abruzzo (ABR), Basilicata (BAS), Calabria (CAL), Campania (CAM), Molise (MOL), Puglia (PUG), and Sicily (SIC) (Figures 1C and 1D; also see Figures S3 and S4 for regional aggregation).

Across Italy, there are, on average, three failures per day affecting electricity utility consumers, but this translates to a failure only once per 11 days within individual regions (range: 2 days in Campania (CAM) and Sicilia up to 56 days in the Valle d’Aosta/Vallée d’Aoste (AOS); Table S3). The total value of lost load is estimated to be 135 m€/year, which translates to an annual value of 2.3 €/person (or less than 1 kWh). These estimates are highly sensitive to the assumed values of lost load, which varies considerably (Table S4); however, even across the available estimates, the average value of lost load per person remains under 6 €/person/year.

Although the total impact on people is small, there are noteworthy performance discrepancies between Italian regions. For example, Figure 1E shows that the annual value of load lost ranges from 0.19 €/person in the AOS (0.07€–0.44€/person) up to 6.34 €/person in CAM (2.3€–14.66€/person). In terms of failures per capita, performance is over five times better in Emilia-Romagna (EMR) as compared to Molise (MOL), Trentino-Alto Adige (TAA), and AOS (Figure 1F). Besides revealing substantial regional differences, Figures 1E and 1F and Table S3 also highlight the sensitivity of assessing equity claims with respect to the metrics used. For example, CAM requires the most support according to the total value of lost load. However, the frequency of outage instead suggests concentrating on AOS and Liguria (LIG).

Considering power system performance (Figures 1E and 1F) with respect to social and infrastructure vulnerabilities to power outages (Figures 1C and 1D) also reveals that poorer power system performance correlates with regional vulnerabilities. Regions in the South of Italy collectively have a higher frequency of failures per capita than their more northern counterparts (Figure 1F); this reinforces existing community and infrastructure vulnerabilities (Figures 1B and 1C). Considering broader geographic partitions confirms that power system performance is worst in the South, which accounts for 48% of all recorded failures in the dataset (5,664/11,831) and hosts the second-highest rate of failures per capita, only behind Sardegna (Figure S3). Notably, the South is also the mainland region with the highest community vulnerability to power outages (Figure S4), endemic inequalities,^22^^,^^26^ and some types of natural disasters.^27^ In other words, lower power system performance in the South reinforces existing inequalities. As such, the worse-off power system performance and high community vulnerability indicate that improving power system performance in the South is relevant for improving equitable power delivery.

Results show that only 10% of failures account for 97% of energy not served (Figure S5), highlighting that extreme events have the greatest impact toward impeding utility electricity delivery. Moreover, the susceptibility to extreme events appears to vary between regions and across (Figure S6). Although full exploration of extreme events is outside the scope of the present work, these results indicate the disproportionate effect of large events on the system and underline the importance of emergency preparedness in assuring equitable power delivery.

### Drivers of power system failures

We identify the causes for the differences in annual failure frequency per region using regression analysis (see quantification and statistical analysis). Here, we include predictor variables where there is a working hypothesized relationship between the predictor variable and the output variable. Namely, previous work has established weather, component type, and community vulnerability to all be relevant to power system performance. Long-run data establishes relationships between components and weather conditions with failure rates,^20^^,^^28^ whereas previous studies link social and environmental vulnerability to worse power system performance^7^^,^^9^^,^^15^^,^^29^ (see characterizing community and infrastructure vulnerability). We adopt continuous and categorical predictor variables to account for these factors within a regression. The continuous variables include regional vulnerabilities (CVI and IVI) and historical weather patterns in wind, run-off from rain and snow, and temperature. We consider these weather variables as they are all known to contribute to power system failures^30^^,^^31^ and correlated with other weather measurements.^32^ Component class is provided as a categorical variable, with 70 kV as the reference value (see Tables S4 and S5 for model formulation).

**Figure 2:**
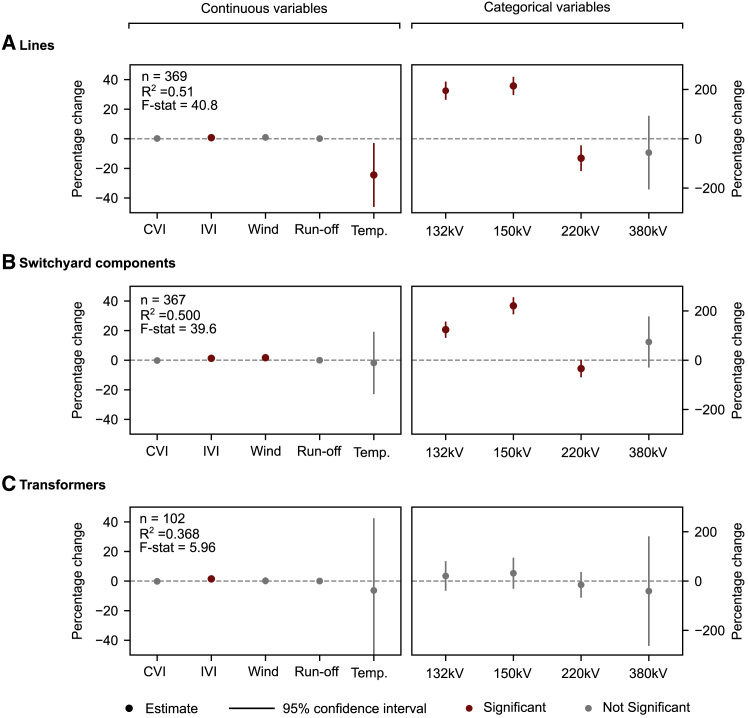
Regression results for failure frequency per capita Regression results for failure frequency per capita against predictor variables considering (A) lines, (B) switchyard components, and (C) transformers. Dots indicate mean estimates and gray bars show the 95% confidence interval. Significant values indicated considering a 95% confidence interval and *p* values <0.05. For continuous predictor variables (left-side panels), the *y* axis shows the percentage change in failure frequency per capita versus a 1% change in the predictor variable. For categorical predictor variables (right-side panels), the *y* axis shows the percentage change in failure frequency per capita versus a ref. 70 kV component. Greater absolute percentage changes in the predictor variables indicate a larger influence on the output variable, the frequency of failure per capita. Reference voltage level: 70 kV. Complete regression results, variance inflation factors, and comparison to an alternative model specification are provided in Tables S6–S10. For details on interpretation, see quantification and statistical analysis. See also Figure S2 and Tables S5–S13.

Figure 2 shows summary results for the regression analysis (see Tables S7–S9 for complete regression results). For continuous predictor variables (left-side panels), the *y* axis shows the percentage change in failure frequency per capita versus a 1% change in the predictor variable. For categorical predictor variables (right-side panels), the *y* axis shows the percentage change in failure frequency per capita versus a ref. 70 kV component. The larger percentage changes in the predictor variables (either negative or positive) indicate a stronger effect on failure frequency per capita. For example, temperature is the most influential continuous predictor variable for failure frequency in lines (Figure 2A). The moderate R^2^ values (shown in the upper left corner of each panel) show that the linear regression models only have partial explanatory power, which we attribute to a high degree of randomness regarding component failure: 27% of all failures in our dataset occur without cause. Including additional predictor variables could also potentially increase the model’s explanatory power. However, information regarding potentially relevant predictor variables like component brand, operating budget, and exposure to the distribution grid are unavailable. Moderate explanatory power could also be caused by omitted non-linear relationships. However, this risk is limited given that variance inflation factors are low across all variables and tests (Table S10).

Importantly, CVI is never a significant predictor of failure frequency. So, although we observe disparate outcomes in terms of impact in the Italian high-voltage power system, we fail to detect any evidence that these disparate outcomes are the direct result of bias or discrimination in system operation. Instead, the important predictor variables of failures in the Italian high-voltage power system are all linked to IVI, weather (wind and temperature), and voltage level. However, overlooking the contribution of infrastructure vulnerability in driving power system performance may lead to the incorrect conclusion that there is system bias: omitting the IVI from the regression analysis suggests that CVI is a significant predictor (Tables S11–S13). These results confirm findings from previous work^29^ stipulating the crucial need to fully consider environmental and infrastructure conditions to accurately determine the causes of subpar performance.

The relative importance of predictor variables varies component to component (Figure 2), which is expected based on the technology-specific failure mechanisms and operating requirements. However, the results do show consistent trends. First, IVI is a positive and significant predictor for all components. This result suggests that there are inherent infrastructure factors affecting power system failures. Notably, these infrastructure factors are shaped by human development and, therefore, within the realm of control, albeit perhaps not by power system operators themselves. For example, road density may affect the ability of repair crews to access damaged components. Importantly, infrastructure vulnerability is always a more relevant predictor than community vulnerability, which is not a significant predictor for any component (see also Tables S7–S9 for numeric results). The relevance of local temperature (for lines, Figure 2A) and windiness (for switchyard components, Figure 2B) also substantiate theoretical expectations and emphasize the value of considering environmental factors (natural and anthropogenic) in analyzing power system failures.

An unexpected finding is the relevance of component voltage level to predicting failure frequency. Components rated at 132 kV and 150 kV are associated with failure frequencies multiple times greater than their 70 kV reference counterparts when all other predictor variables are kept constant (though the result is not significant for transformers; Figure 2C). Generally, we would expect lower voltage components to be more subject to failure, given their closer relative proximity to operational issues stemming from the distribution grid. However, our results show that the assumption is not uniformly valid. The greater failure frequencies observed at 132 kV and 150 kV suggest that components at this level may be overlooked sources of failure within the high-voltage electricity network. One explanation for this phenomenon is that 132 kV and 150 kV networks tend to be overloaded in the vicinity of large cities^33^ and may consequently fail at a higher rate than otherwise expected (see Figure S8 for a spatial distribution of substations based on the voltage level).

### Opportunities for improving regional system performance

The South of Italy has the second-highest community vulnerability to electrical power outages yet simultaneously experiences the country’s worst absolute transmission power service (Power system performance assessment). Finding measures to improve power system performance in the South is, therefore, especially valuable. Building on previous results (Drivers of power system failures), we investigate the failure patterns in the South in more detail to identify opportunities for correcting performance deficiencies. To do so, we rank substations according to the frequency of reported failures and identify the worst 5% of substations (past the 95^th^ percentile). We refer to these substations as “worst-performing substations”. We then analyze component failures according to the main cause of failure, voltage level, component, and ownership between these worst-performing substations and all others to identify opportunities for improving regional system performance.

**Figure 3:**
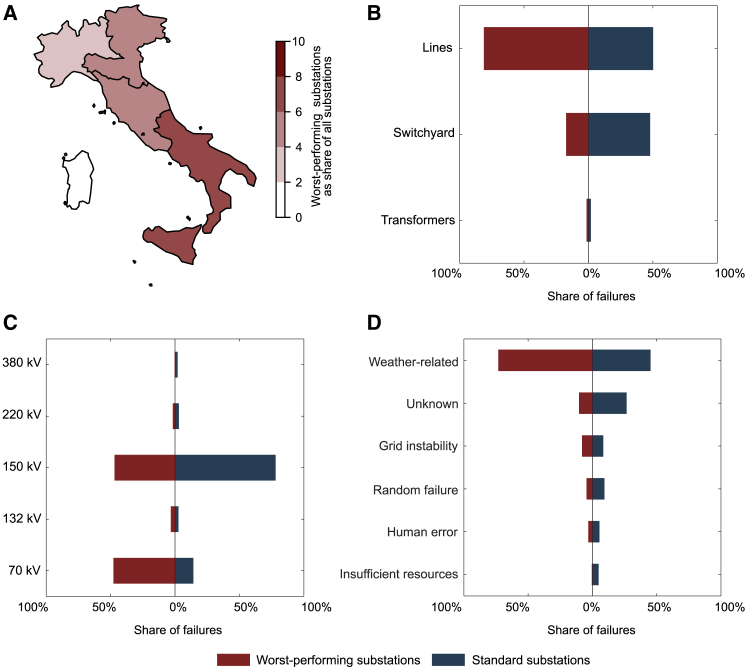
Substations in the South of Italy (A) The prevalence of worst-performing substations across Italy, as measured by frequency of failure, with the highest concentration occurring in the South. The remaining panels show the failure patterns in worst-performing substations versus all other substations for the South of Italy according to (B) affected component, (C) voltage level, and (D) main cause of failure. Note that the distribution of failures per voltage level shown in (D) differs from the overall distribution of components per voltage level listed in Table S15. See also Figures S7 and S8.

We find that the South is neither more exposed to certain types of failure nor to the effects of particularly significant outages (Figures S5 and S7). However, the South has a higher-than-average share of poorly performing substations, hosting 60% of the worst-performing substations. This share translates to nearly 9% of all substations in the South (Figure 3A). The over-concentration of worst-performing substations in the South is statistically significant and consistent even when considering different thresholds for what is considered a worst-performing substation (Figure S9; Table S14). Combined, the worst-performing substations in the South account for 19% of the energy not served in the South and 9% of all energy not served in all of Italy. This finding suggests that subpar performance at specific point locations is responsible for disparate power system performance for utility electricity consumers. By extension, improving performance at these “hot spots”^34^ is also key to providing equitable power system performance.

Failure patterns at worst-performing substations vary considerably compared to what would be expected based on failure patterns at all other substations. In the worst-performing substations, a far greater share of failures occurs in lines (Figure 3B), in 70 kV components (Figure 3C), and due to weather-related causes (Figure 3D) as compared to other substations. These patterns are linked, as 36% of all failures in worst-performing substations are weather-induced failures in 70 kV lines. As is the case for all of Italy, 99% of weather-related failures at worst-performing substations affect lines; however, there are much higher failure rates due to weather-related events in worst-performing substations compared to the entire dataset (73% versus 56% of failures). The relatively higher rates of weather-related failures at outlier substations point toward a lack of proactive measures by the asset owners to counter weather-related failures, especially as we detect no other meaningful regional differences in failure patterns or system structure (Table S15). Potential corrective measures include adjusting operating procedures ahead of extreme weather events and clearing line-ways of potential debris (e.g., tree branches) more frequently. Although the consideration of lightning-induced failures remains outside the scope of this work, transmission system planners may also consider compact and multi-circuit line designs, which can minimize lightning-induced overvoltages.^35^^,^^36^

**Figure 4:**
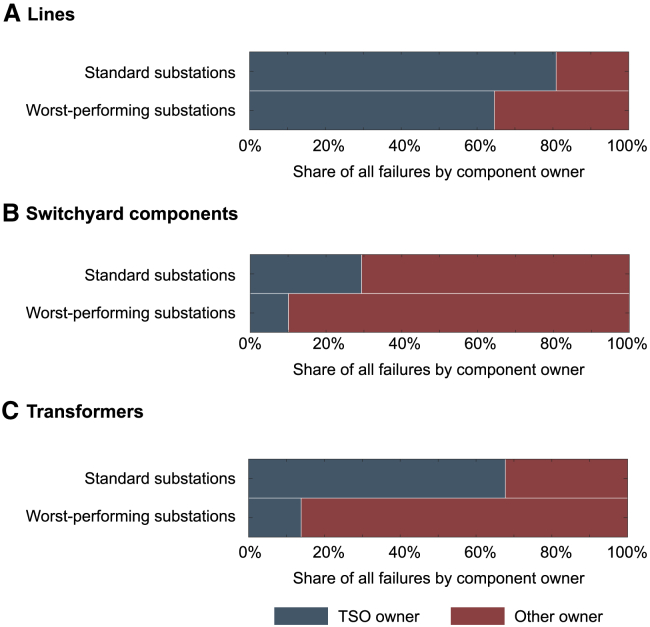
Failures by component, owner, and substations Worst-performing substations attribute a higher share of failures to components owned by non-TSO actors than standard substations. This phenomenon is apparent for lines (A), switchyard components (B), and transformers (C). See also Figure S7.

Figure 4 shows that higher rates of failure can be linked to component owners, with the worst substations attributing a greater share of failures to non-TSO-owned components than all other substations. This finding suggests that the performance of assets under TSO ownership is more consistent than those owned by other power system participants. Potential causes for these discrepancies could be differences in organizational maintenance practices, the components themselves (e.g., purchased from different suppliers with accordingly varying standards), or in managerial composition.^37^ Further investigation is needed to provide conclusive findings and recommendations.

## Discussion

In centralized electricity systems, high-voltage power systems are the backbone of power delivery and, therefore, crucial to delivering fair power service. Here, we address a research gap in the energy equity space by studying historical system performance in the Italian high-voltage electricity grid. We find that, although the Italian high-voltage electricity system is highly reliable, inequities still exist. The total impact of lost power on consumers is small compared to the inconvenience caused by the distribution system.^38^ However, more socially vulnerable regions experience worse power system performance than their less vulnerable counterparts. Nonetheless, we find no evidence that the performance discrepancies in the most vulnerable regions are due to bias. Instead, the disproportionate impacts can be explained by differences in prevailing weather conditions, inherent infrastructure vulnerabilities, and subpar performance in some technical components.

Our results reinforce the importance of (1) accounting for environmental factors (natural and human-induced) when analyzing power system outages to suggest appropriate corrective strategies^34^ and (2) avoiding to pre-emptively conclude that unequal power systems performance is the result of active discrimination.^29^ In addition, our work also contributes to the literature in identifying energy inequities in Europe and in establishing the high-voltage electricity system as a relevant infrastructure to enabling energy equity. We emphasize that even small differences in power systems quality between regions can be highly relevant to the individuals who experience outages, especially those who are already more vulnerable due to prevailing health, social, and economic characteristics. Investing in vulnerability reduction, or individuals’ capacity to be resilient to power system outages, would help mitigate the negative personal effects of power loss.

Extreme events and poorly performing substations drive unequal power system performance. It follows that better preparations for extreme events and resolving performance issues at these worst-performing substations represent means of directly supporting more equal power performance. Future work should clarify whether specific operations and maintenance practices explain why such a small group of events and substations account for such a large share of failures. Standardizing maintenance and operations procedures between operators would be one route to reduce recovery time and failure frequency. We also recommend continued monitoring to determine whether performance improves over time; if not, the regulator could mandate new operators or operating conditions on the problem substations.

Our results agree with expectations^30^ that ownership type, weather conditions, and component type affect the frequency of failure. Interestingly, there is a discrepancy between the voltage levels most associated with failures across all of Italy (132 kV and 150 kV) and those in the worst-performing substations (70 kV). Our findings suggest that 132 kV and 150 kV components may generally be overlooked in discussions of power systems reliability: these components are neither directly exposed to cascading issues from the low-voltage grid nor as impactful in terms of single-event power losses as 220 kV and 380 kV components. Our results substantiate previous work suggesting that the performance of 132 kV and 150 kV components may disproportionately affect performance in the high-voltage grid, the cause of which may be congestion in and around large cities.^33^ Conversely, the higher failure rates of 70 kV components in the worst-performing substations can largely be tied to weather-induced line failures, suggesting a need for more proactive against weather-related failures by asset owners.

Infrastructure vulnerability, as measured by the IVI, is an even more consistent predictor for failure frequency than weather variables. This result agrees with previous work highlighting the importance of environmental factors in understanding power system failures.^29^ Nevertheless, they extend the realm of consideration to a broader range of environmental factors, including human-influenced designs, like road connectivity. The vulnerability indices applied here, the IVI and CVI, extend the existing literature on vulnerability indices by focusing on power systems and are easy to implement because they rely on openly available data. However, future work should seek to develop a more generalized understanding of power system vulnerability and driving environmental factors (e.g., flood proneness degree). This work should also establish how these factors differ in importance between transmission (high-voltage) and distribution (low-voltage) systems, if at all.

We also find that shared responsibilities for power system operation and maintenance may inadvertently lead to disparate power system performance. In the case of Italy, this is apparent in the over-concentration of power system failures in the South. While the responsibility for managing high-voltage electric power systems may be shared between national TSOs and other actors for historical or economic reasons, future work should deepen the understanding of how ownership structures and organizational practices can lead to subpar power systems’ performance. A detailed comparison of operations and maintenance practices, purchasing standards, and managerial motivations^33^ is needed to identify deficiencies.

Altogether, our work confirms the relevance of considering large-scale, high-voltage electric power systems to achieve equitable power service, even in contexts with highly reliable systems. Nonetheless, energy systems are highly interdependent and interconnected; as such, a fuller understanding of power system impacts requires investigating power system outages across multiple spatial contexts (local, regional, and national) simultaneously, as well as considering environmental conditions at the time of outage (e.g., heat wave, daytime/nighttime). In addition, considering a wider scope of failures, i.e., beyond those affecting utility consumers, would facilitate a fuller characterization of the social and economic impacts of high-voltage power system failures on Italian society.^18^ This multi-scalar and multi-impact approach would help elicit the most important and efficient opportunities for achieving equitable energy delivery in standard and disaster operational settings.

### Limitations of the study

This study is primarily limited in how it characterizes the lived experience of enduring power system failure. This shortcoming is present in four main regards. First, we assume that failures affecting utility-connected substations have a uniform value of lost load corresponding to median estimates for the Italian residential sector. In reality, utility providers also serve other types of end consumers, e.g., restaurants and schools. This simplification is necessary as we lack sufficient information to disaggregate utility-level electricity demand into different demand segments. Aggregating segments is acceptable given that the difference between segment-specific values of lost load are smaller^39^ than the uncertainty surrounding absolute estimates for values of lost load (Table S4); however, the aggregation also implies that the absolute value of lost load we report is likely an over-estimate of the actual value of lost load experienced by individuals. Second, we restrict our impact and vulnerability analysis to the NUTS2 regional scale. Working with this scale is valuable in understanding how entire regions are affected by power outages, in facilitating data collection, and in supporting statistical analysis. The scale is also commensurate with the scale of the Italian power transmission system, i.e., the transmission system is present in every NUTS2 region but not necessarily in smaller geographical boundaries (e.g., NUTS3). Nonetheless, only considering the regional scale assumes a collective experience for all individuals within the NUTS2 region and overlooks how individuals might experience an outage. Third, our CVI and IVI metrics are static, though our analysis covers 10 years. Ideally, our analysis would consider temporally evolving metrics to reflect changing regional vulnerabilities; however, this limitation is necessary due to data unavailability for all years. Finally, we do not consider the disutility to the consumer due to the specific environmental conditions at the time of the power outage. For instance, a 30-min power outage at 02:00 may have less impact on the average person than at 14:00. Likewise, a power outage when the outdoor temperature is 20°C may be less burdensome than when the outdoor temperature is −20°C. Considering the precise socio-environmental contexts in which power outages occur may lead to different recommendations regarding which preventative and corrective actions to take.

## Resource availability

### Lead contact

Further information and requests for resources and reagents should be directed to and will be fulfilled by the lead contact, Giovanni Sansavini (sansavig@ethz.ch).

### Materials availability

This study did not generate new materials.

### Data and code availability

Datasets used in this work have been deposited at https://gitlab.ethz.ch/andrejst/rre-datasets.git and are publicly available as of the date of publication. This paper does not report original code. Any additional information required to reanalyze the data reported in this paper is available from the lead contact upon request.

## Acknowledgments

The research published in this report was carried out with the support of the 10.13039/501100005380Swiss Federal Office of Energy as part of the SWEET PATHFNDR and 10.13039/100023753SWEET EDGE consortia. The authors bear sole responsibility for the conclusions and results presented in this publication. We thank Fatima Safraz, Fiona Kriwan, Francesco De Marco, Ciyu Qin, and Laya Das. We also thank the participants of the ESREL 2024 conference and Prof. Dr. Hiba Baroud for their input on earlier versions of this work. Finally, we also thank the two anonymous reviewers for their time, insights, and helpful suggestions.

## Author contributions

Conceptualization, K.E.L. and A.S.; methodology, K.E.L. and A.S.; investigation, K.E.L. and A.S.; writing—original draft, K.E.L.; writing—review and editing, A.S., B.G., and G. S.; funding acquisition, B.G. and G. S.; supervision, B.G. and G.S.

## Declaration of interests

The authors declare no competing interests.

## Declaration of generative AI and AI-assisted technologies

During the preparation of this work, the authors used Grammarly for editing support. After using this tool or service, the authors reviewed and edited the content as needed and take full responsibility for the content of the publication.

## STAR★Methods

### Key resources table

**Table:** 

REAGENT or RESOURCE	SOURCE	IDENTIFIER
**Deposited data**		

Processed data used in the study	Various	https://gitlab.ethz.ch/andrejst/rre-datasets.git
Data for remoteness indicator	Eurostat^40^	Regional population living in or near rural areas
Data for vegetation indicator	Eurostat^40^	Regional forest and shrubland area
Data for connection density indicator	Eurostat^40^	Regional motorways, railroads, and roadways
Data for mountainous population	Eurostat^40^	Regional population living in mountainous areas
At-risk-of-poverty rate by NUTS2 regions	Eurostat^41^	Eurostat: tgs00103
European Blackouts Database	ETH Zurich^20^	RRE Datasets: 02_National_dataset
Income of households by NUTS 2 regions	Eurostat^42^	Eurostat: nama_10r_2hhinc
Italian substations	TERNA^43^	Censimento Utenti
Italian transmission grid characteristics	CIGRE^44^	Italian Power System
Indexed time for essential services	Istat^45^	Istat: Indice di accessibilità ai servizi essenziali (CFI_MUN)
Population by NUTS3 region	Eurostat^46^	Eurostat: demo_r_pjangrp3
Quality of transmission system reports	TERNA^15^	Rapporto annuale qualità e altri output del servizio transmissione
Share of healthy population	Istat^47^	Istat: Health status - regions and type of municipality
Share of population with low education	Istat^45^	Istat: Popolazione di età compresa fra 25 e 64 anni con titolo di studio non oltre la licenza di scuola media inferiore o di avviamento professionale (CFI_MUN)
Weather data	Copernicus Climate Change Service^32^	C3S: ERA5 hourly data on single levels from 1940 to present

**Software and algorithms**		

MATLAB	MathWorks^48^	https://ch.mathworks.com/products/matlab.html
Jupyter Notebook	Project Jupyter^49^	https://jupyter-notebook.readthedocs.io/en/stable/
Python 3.11.9	Python Software Foundation^50^	https://www.python.org/

### Method details

#### Geographic scope and scale

Our analysis focuses on Italy. The combined availability of failure data in the Italian power system (see power system failure data in method details) and abundant socio-economic data (see key resources table) make the country suitable for a data-based energy equity study. However, our methods could equally be applied to other locales if comparable data exists.

The main geographic units we consider are the Italian NUTS2 regions defined by the European Union.^51^ Sufficient information exists at this level of analysis to collect detailed statistical data and to assign electrical substations to regions. Working at a more refined geographic scale would facilitate a more nuanced analysis of individual communities. Furthermore, it would reveal possible vulnerability “hot spots”^34^ lost in the aggregation process, e.g., the vulnerability of a small, poor community if it is surrounded by larger, wealthier communities. However, insufficient socio-economic data exists at a more refined spatial scale (e.g., NUTS3).

For parts of our analysis, we also consider power system performance by large geographic areas. These areas are based on the NUTS1 classification of the European Union,^13^ except that we consider the island of Sicily part of the South area (Figures 1A and S1; Table S1). Merging Sicily and the South is justifiable from a power systems perspective, as the Sicilian transmission is functionally connected to the mainland. Considering multiple geographic scales is beneficial as it allows us to confirm patterns observed in the regional analysis (Power system performance assessment in Results and Figures S3 and S4).

#### Power system failure data

In this study, we use a curated dataset of outages from the Italian transmission system originally reported by the Italian transmission system operator, TERNA, in their yearly “Quality of transmission service” reports and initially analyzed in Stankovski et al.^20^ The data includes all failures that impact the electric transmission system, including failures that originate from the distribution grid. Each data observation includes information about the affected component, duration, cause, and energy not served. The location of component failures is recorded at the nearest substation. This information includes the name of the substation, voltage, type, and component owner. An extended description of the data features, cause of failure, and types of components is provided in Bednar et al.^14^ To complement the existing dataset, we take three additional post-processing steps. First, we associated failure location with the 20 NUTS2 regions of Italy by identifying the coordinates of the recorded substations. Altogether, we process information for 3,765 unique substations. Second, we consider only a subset of the original dataset to focus our analysis specifically on outages affecting electricity utility consumers, i.e., we exclude outages that affecting manufacturing facilities, railways, water utilities, and other consumers.^43^ Outages affecting these other types of electricity consumers undeniably have adverse social effects, but estimating the cost to society is more complicated and, thus, left to future work. Third, we characterize annual regional weather based on data from the Copernicus Climate Change service^32^ (key resources table). Doing so allows us to control for the influence of weather on failure likelihood.

#### Power system performance of regions and substations

We characterize regional power system performance at the NUTS2 region using the power system failure data (see power system failure data in method details) and the outage metrics in Table 1. Multiple metrics are necessary to understand power system performance and related social impacts over an extended time horizon as they imply different social impacts. Namely, the failure frequency and total energy not served describe different aspects of inconvenience.

#### Characterizing community and infrastructure vulnerability

We characterize regional vulnerability to power outages considering socio-economic and infrastructure-related factors. On one hand, considering socio-economic factors alludes to the inherent vulnerabilities of individuals.^12^ On the other hand, considering regional infrastructure provides an overview of structural factors that might make entire places vulnerable to the impacts of a power outage.^52^ Vulnerability to infrastructure failure is often summarized via indices, which can offer an efficient summary of multiple vulnerability factors and facilitate constructive discussion between diverse stakeholder groups.^53^ However, working with indices may also be challenging as indices require extensive data and may be difficult to validate.^25^ There are also gaps in terms of what indicses are available: numerous indices describe vulnerability to natural disasters, but fewer options are available for describing vulnerability to human-induced events.^25^

Our approach of delineating social and geographic factors follows previous work showing that controlling for geographical factors can affect whether socio-economic factors are relevant predictor variables of power system performance.^29^ We summarize all community-related vulnerability indicators into a community vulnerability index (CVI) and all infrastructure-related indicators into an infrastructure vulnerability index (IVI). Both the CVI and IVI have a range of 0–1, with higher values indicating higher vulnerability.

The CVI measures community vulnerability to the effects of power blackouts. Existing literature suggests an extensive list of possible indicators for community vulnerability^25^^,^^26^^,^^54^; however, these indicators neither specifically describe vulnerability to power outages nor are the constituent components necessarily transferable to an Italian context. For example, American literature often considers race and ethnicity in estimating community vulnerability, an approach based on historical discrimination in the United States.^55^ In addition, while many possible indicators could be used,^56^ research has shown that patterns of community vulnerability can be effectively represented using a small (around five) subset of explanatory factors.^29^ In previous work,^29^ factors included wealth, poverty, elderly populations, access to civil agencies, and non-native language speakers. We build our CVI considering the first four factors, plus the share of people in poor health and with low education. We consider these latter two factors to account for populations physically vulnerable to the loss of electricity service^12^^,^^57^ and in consideration of the Italian National Institute of Statistics (Istat) definition of municipal fragility.^45^ Altogether, our CVI is constructed based on the following six metrics.

(1) At-risk-of-poverty rate: percentage of the population at risk of poverty or social exclusion.
(2) Wealth: average income per inhabitant (€). We use income as a proxy for wealth in the absence of suitable wealth data.
(3) Mean access to essential services: indexed time to reach essential services.
(4) Population dependency index: the share of the non-working population versus the share of the working population.
(5) Share of population with low education: percentage of the population with only primary education.
(6) Share of healthy population: percentage of people with perceived good or very good health.

We introduce the concept of an infrastructure vulnerability index (IVI) to capture the inherent structural factors of a region that may contribute to both power system performance and the impact of an outage on individuals. Here, we build our IVI using the following five indicators, which collectively describe potential structural challenges stemming from under-investment, aging infrastructure, and complexity of accessing failed components due to difficult terrain and remoteness.

(1) Regional gross domestic product (${GDP}_{i}$) per capita: an indicator of regional wealth. We assume that regions with lower GDP per capita have lower disposable income to invest in the power system infrastructure, especially on the distribution side. We use the average regional GDP from 2013 – 2022.
(2) Connection density: We derive this indicator from the Eurostat indicators for motorways, railroads, and rural roads in a region (Equation 1). Regions with lower connection density are less connected, which can result in longer response times from the repair teams.

(Equation 1) $${CD}_{i}=\frac{l_{motor}^{i}+l_{rail}^{i}+l_{road}^{i}}{A_{i}}$$

where ${CD}_{i}$ is the connection density indicator for region i in km/km^2^, $A_{i}$ is the total area of region i in km^2^, $l_{motor}^{i}$, $l_{rail}^{i}$ and $l_{road}^{i}$ are the kilometers of motorways, railways, and local roads in the region, respectively.^58^.

(3) Vegetation indicator (${VI}_{i}$): Percentage of the area covered by forests and shrubland. Repair teams can have difficulties carrying specialized equipment across areas covered in vegetation, prolonging the repair time. Additionally, vegetation growth is a frequent cause of short circuits, potentially increasing the number of failures. We derive this indicator as a summation of the forestry and shrubland indicators published by Eurostat^40^ (key resources table).
(4) Mountainous population indicator: An indicator for whether 50% of the population lives in mountainous areas.^40^ Mountainous regions can be challenging to traverse, potentially making failed components more difficult to access, especially during extreme weather conditions. As the raw data is published on a sub-regional level (NUTS3) as a binary (0 or 1), we aggregate the data to a regional (NUTS2) level and use the population^46^ as weights to calculate the indicator (Equation 2):

(Equation 2) $${MP}_{i}=\sum_{j}\frac{P_{j}}{P_{i}}\cdot m_{j}$$

where ${MP}_{i}$ is the mountainous population indicator of region i, $P_{i}$ is the population of region i, $P_{j}$ is the population of the sub-region j, and $m_{j}$ is a binary value that indicates whether at least 50% of the population in the sub-region lives in a mountainous area.

(5) Remoteness indicator: An indicator of the percentage of the population living in or near rural areas. This index is a proxy for the remoteness of the infrastructure itself. Because of their lower populations, rural areas tend to have lower priority than urban centers for benefiting from preventive maintenance and during outage recovery. Like the indicator for mountainous population, we derive the remoteness factor by aggregating data from the sub-regional level to the regional level, using the population^46^ as weights (Equation 3; key resources table).

(Equation 3) $${RI}_{i}=\sum_{j}\frac{P_{j}}{P_{i}}\cdot r_{j}$$

where ${RI}_{i}$ is the remoteness indicator of region i, $P_{i}$ is the population of region i, $P_{j}$ is the population of the sub-region j, and $r_{j}$ is describes the remoteness of the population in sub-region j. $r_{j}$ ranges from 0-1, or from predominantly urban to predominantly rural, in increments of 0.25.

We use these five indicators to introduce the concept of an IVI; however, the suitability of the indicators may vary according to location. For example, considering the share of the population living in mountainous areas is irrelevant in a country without mountains. Conversely, other environmental factors may be more relevant in other areas, e.g., the share of the population living within flood plains. The concept of the IVI will be further developed and tested in our future work.

To avoid unit-based biases, we scale the components of the CVI and IVI according to minimum and maximum values (Equation 4). To control the fact that lower GDP, connectivity, and shares of healthy people indicate higher vulnerability, we apply a logical negation to the indicators for regional wealth, connection density, and the share of healthy people (i.e., we take 1 minus the normalized value). Finally, we build the composite IVI by taking the arithmetic mean of the indicators (Equation 5):

(Equation 4) $$X_{k,scaled}^{i}=\frac{X_{k}^{i}-\min\left(X_{k}^{i}\right)}{\max\left(X_{k}^{i}\right)-\min\left(X_{k}^{i}\right)}$$

(Equation 5) $${VI}_{i}=\frac{\sum_{k=1}^{N_{i}}X_{k,scaled}^{i}}{N_{i}}$$

where $X_{k,scaled}^{i}$ is the scaled form of the indicator $X_{k}^{i}$ for region i, $N_{i}$ is the total number of indicators considered, and ${VI}_{i}$ is the vulnerability index (CVI or IVI) for region i. We use an arithmetic mean rather than non-compensatory methods because the number of indicators is relatively small, and the complexity of interactions between the indicators makes it difficult to assign weights without introducing bias.^19^^,^^20^ However, relying on the arithmetic average is also a limitation of the study, as non-compensatory measures can potentially improve the performance of the indices and reduce correlations between constituent components (Figure S2). The drawback of introducing weights is the need for a case-by-case calibration aligned with context-specific underlying theoretical vulnerability frameworks.^53^

We again highlight that the CVI and IVI are developed with respect to vulnerability to power outages; however, they include some vulnerability features present in other, more general indicators (Figure S2). Nonetheless, vulnerability is context-specific, hence the benefit of using context-specific vulnerability indicators.^25^

#### Calculating the impact of power system outages

We estimate the overall societal impact of a power system outage in euros*.* Describing the impact of a power outage in monetary units rather than only in energy loss (MWh) embeds the value of the intended end-use into the assessment process. Estimating the societal impact of lost power in monetary units is common practice in forward-looking reliability assessments.^59^ Such an approach enables the analysis of the impacts of real-world events.^60^ Post-analysis of real-world outages also commonly relies upon outage duration^10^^,^^11^^,^^15^; however, we lack sufficient data to estimate the outage duration experienced per person. As such, we measure outage impact in terms of cost per person (€/person) to facilitate an inter-regional comparison and maintain a focus on impacts to individuals.

We estimate the impact of lost power on a per capita basis by first multiplying the lost load (MWh) listed in the power system failure data by the value of lost load (VoLL) for residential consumers in Italy and then dividing the total impact by population. The VoLL describes the price that end-users would be willing to pay to avoid an outage and is often expressed in units of cost per energy lost or cost per outage duration (e.g., €/kWh or €/hour).^61^ The VoLL can be estimated using a variety of methods, including surveys, revealed choice experiments, and proxies based on retail electricity prices, wage rates, and other economic indicators.^61^ However, the VoLL does not account for the macroeconomic losses stemming from a power outage.^61^ VoLL estimates may vary according to the method in which estimates are created, as well as across consumer segments, location, and outage scenario (e.g., a power loss at midday versus during the evening peak; a 2-min loss versus a 60-min loss).^62^ Nonetheless, national reliability standards require only a single VoLL per consumer segment.^59^ Here, we consider the median value for the VoLL in the Italian residential segment as determined in previous work (see Table S4). The Italian residential VoLL is the sixth highest among European Union Member States, indicating a comparatively high valuation on leisure time within the country compared to other Europeans.^63^

### Quantification and statistical analysis

We perform three main types of statistical analysis: a correlation analysis, Fisher’s exact test, and weighted linear regression. We perform a correlation analysis to quantify the relationship between constituent variables in the CVI and RVI (Figure S2) and between the CVI, RVI, HDI, and FI (Figure 1). Fisher’s exact test is used to determine the association between categorical predictor variables.^64^ Specifically, the test relies on count data for binary outcomes and tests whether the number of positive outcomes differs significantly between categories. In this study, we apply Fisher’s exact test to compare the share of failures recorded by owner and substation type across different component types (Figure 4).

We perform a weighted linear regression (Equation 6) to understand the relationship between potential causes of power system failure and the nature of the failures (see drivers of power system failures in results):

(Equation 6) $$\ln\left(Y_{i}\;\right)=\beta_{0}+\beta_{j}\;\ln\left(X_{j}\right)+\beta_{k}Z_{k}+\epsilon$$

where $Y_{i}$ is the response variable, $X_{j}$ are continuous predictor variables, $Z_{k}$ are categorical predictor variables, $\beta_{0}$ is the intercept, $\beta_{j}$ and $\beta_{k}$ are the set of coefficients, and ϵ is the error term. Each observation describes the annual failure rate with respect to annual average environmental conditions per region and component.

We weigh observations to correct for sample bias: our power system failure data contains only information about components that failed but no information about components that did not fail. Bias correction is essential to the analysis because we investigate aggregate power system performance rather than characterize individual failures (e.g., average duration of a transformer failure). Failing to perform bias correction may lead to erroneous conclusions, e.g., we might conclude that failures are more likely at a given voltage level simply because there are more components at that given level. We weigh observations^65^ according to the inverse proportions of components at a given voltage level based off the known share of lines per voltage level^44^ (Table S15). An alternative strategy to weighted observations is to use imputed values; however, tests during model development suggested that weighting observations is the most appropriate strategy for this study (Table S6).

For ln-ln regressions, the coefficient $\beta_{j}$ describes the percentage change in the response variable expected by a 1% change in the predictor variable.^66^ This result can be shown by exponentiating Equation 6 to obtain Equation 7, then taking the derivative of Equation 7 with respect to X to obtain Equation 8.

(Equation 7) $$Y=e^{\beta_{0}}X^{\beta_{j}}e^{\epsilon }$$

(Equation 8) $$\frac{\partial Y}{\partial X}={\beta_{j}e}^{\beta_{0}}X^{\beta_{j}-1}e^{\epsilon }$$

Dividing the left and right-hand sides of Equation 8 by Y leads to Equation 9. Canceling constant terms $e^{\beta_{0}}$ and $e^{\epsilon }$, and rearranging gives Equation 10, which finally shows how changes in predictor variable X lead to changes in response variable Y.

(Equation 9) $$\frac{\left(\frac{\partial Y}{\partial X}\right)}{Y}=\frac{{\beta_{j}e}^{\beta_{0}}X^{\beta_{j}-1}e^{\epsilon }\;}{e^{\beta_{0}}X^{\beta_{j}}e^{\epsilon }}$$

(Equation 10) $$\frac{\partial Y}{Y}=\beta_{j}\frac{\partial X}{X}$$

For example, $\beta_{j}=0.2$ implies that increasing X by 1% increases Y by 0.2%.^66^

For categorical predictor variables, the percentage change in the response variable can meanwhile be interpreted according to Equation 11:

(Equation 11) $$\%\Delta_{\beta_{k}}=\left(e^{\beta_{k}}-1\right)\cdot 100\%$$

For example, $\beta_{k}=0.5$ implies that changing from the reference category to category k increases the response variable by $\left(e^{0.5}-1\right)\cdot 100\%\approx 65\%$.

Information about each regression is provided within Tables S5–S10. This information includes exact predictor variables, number of observations, and *p*-values (∗: *p*-value <0.05, ∗∗: *p*-value <0.01, ∗∗∗: *p*-value <0.001). All quantitative results are found using MATLAB^48^ and some results are plotted using Python.^50^
