# Supplementary material for: Disparate power transmission performance reinforces Italian social inequities
Source: iScience. 2025 Jun 20;28(7):112953. doi: 10.1016/j.isci.2025.112953 (PMC12270701; doi:10.1016/j.isci.2025.112953)
Supplement: Document S1. Figures S1–S9 and Tables S1–S15 [file mmc1.pdf]

iScience, Volume *28*

Supplemental information

Disparate power transmission performance reinforces Italian social inequities

Katherine Emma Lonergan, Andrej Stankovski, Blazhe Gjorgiev, and Giovanni Sansavini

Supplemental information

This document contains Figures S1–S9 and Tables S1-S15.

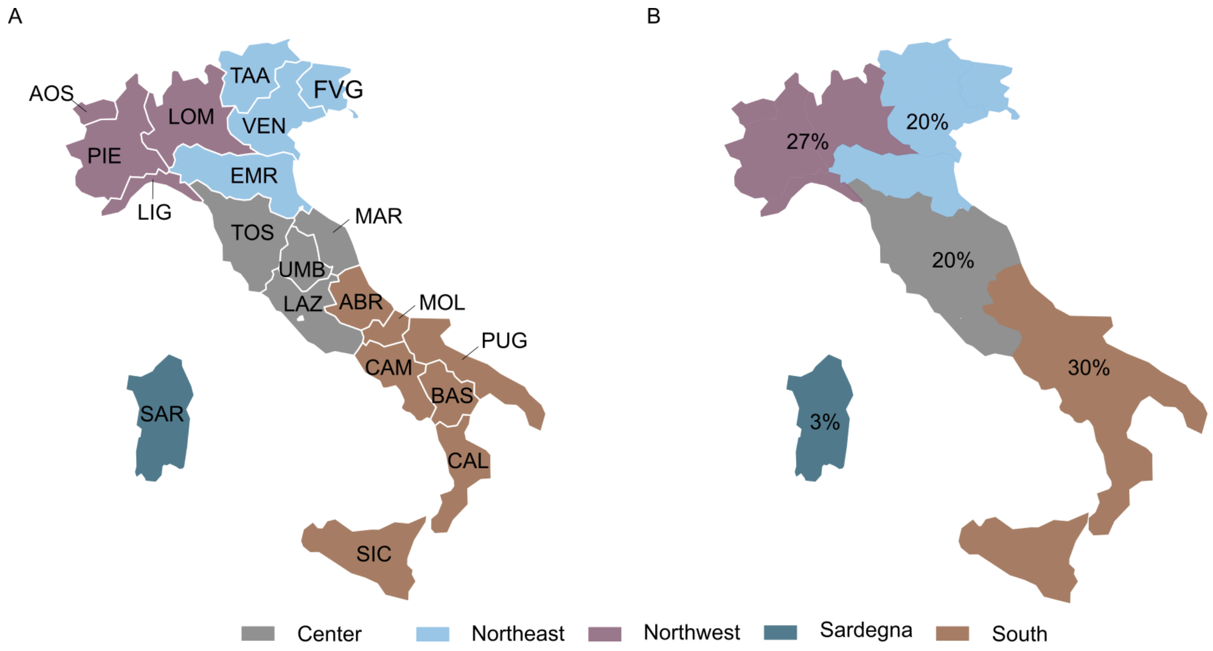

Figure S1. Italian regions by area groupings, related to Figure 1

(A) Italian regions colored by area groupings. (B) Share of population within each region. Area groupings are described in Geographic scope and scale in Method details. Regional abbreviations are listed in Table S1.

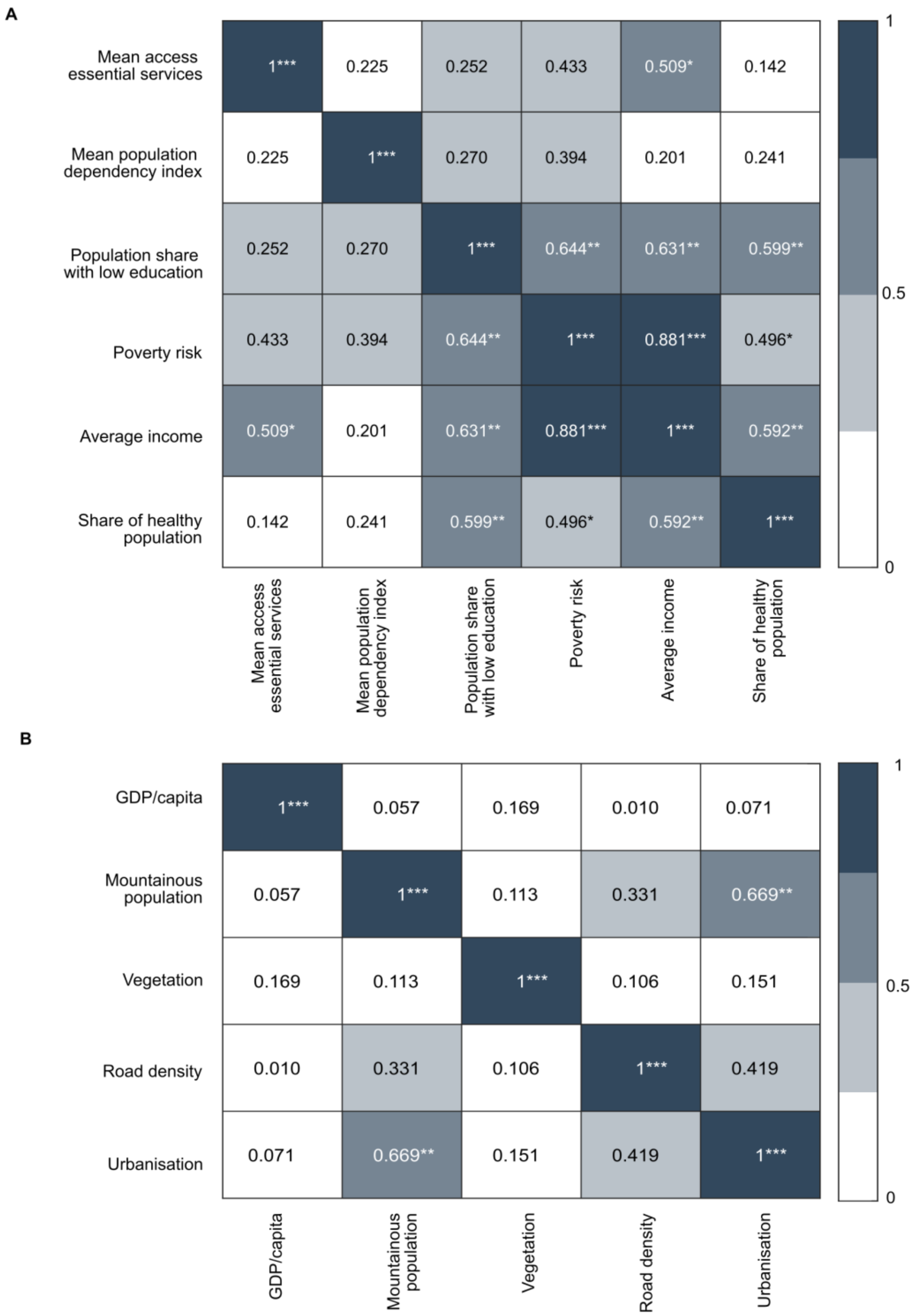

Figure S2. Correlation heatmap between constituent components for the CVI and IVI, related to Figure 2

The figure illustrates the absolute correlations between the components considered within (A) the CVI and (B) the IVI. *: p-value <0.05, **: p-value <0.01, ***: p-value < 0.001. The highest correlation is observed between average income and poverty risk. Although these two factors are highly correlated in our case study, the same is not necessarily generally true: the strength of the correlation can vary according to inequality, local purchasing power, and access to services. Several other variables have moderate-high correlations (0.5-0.7), including the population with a share of low education and average income. correlation. While the construction of the CVI is based on theoretical understandings of community vulnerability and some degree of correlation between indicator components is always to be expected[1], future work should look to revise the formulation to reduce the presence of correlation among constituents components. The components of the IVI are altogether more independent, with only urbanization and mountainous population showing moderate-high (0.67) correlation.

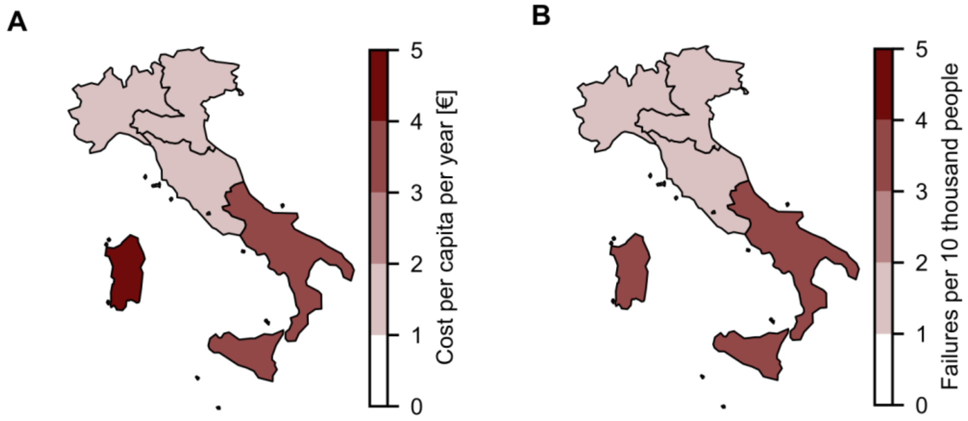

Figure S3. Power system performance across large areas, related to Figure 1

(A) Cost per capita per year and (B) failures per ten thousand people across large areas. Areas correspond to groupings shown in Table S1 and Figure S1. As shown in Figure 1B-C, the performance of the electric power transmission system varies according to what performance metric is considered. South Italy has the worst outcomes in failures per capita; it is also the second-worst performer in terms of terms of cost per capita. Note that some geographic discrepancies in local vulnerability apparent at a regional level (Figure 1D-E) are lost when regions are aggregated to larger geographic units. A similar phenomenon is observable for regional vulnerability, as shown in Figures 1D-E and in Figure S3.

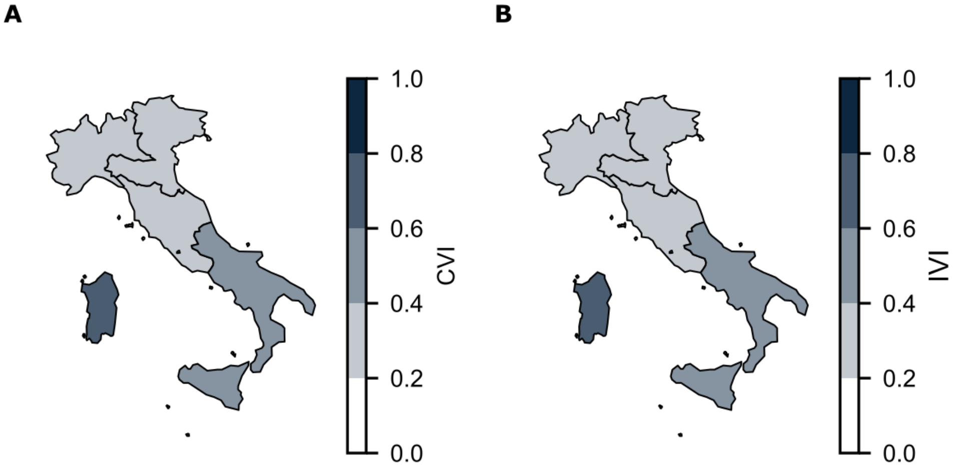

Figure S4. Regional vulnerabilities across large areas, related to Figure 1

(A) Community Vulnerability Index (CVI) and (B) Infrastructure Vulnerability Index (IVI) by large area. Areas correspond to groupings shown in Table S1 and Figure S1. Sardegna is the most vulnerable region according to both the CVI and IVI followed by the South. Note that some geographic discrepancies in local vulnerability apparent at a regional level (Figure 1D-E) are lost when regions are aggregated to larger geographic units. A similar phenomenon is observable for power system performance, as shown in Figures 1B-C and in Figure S2.

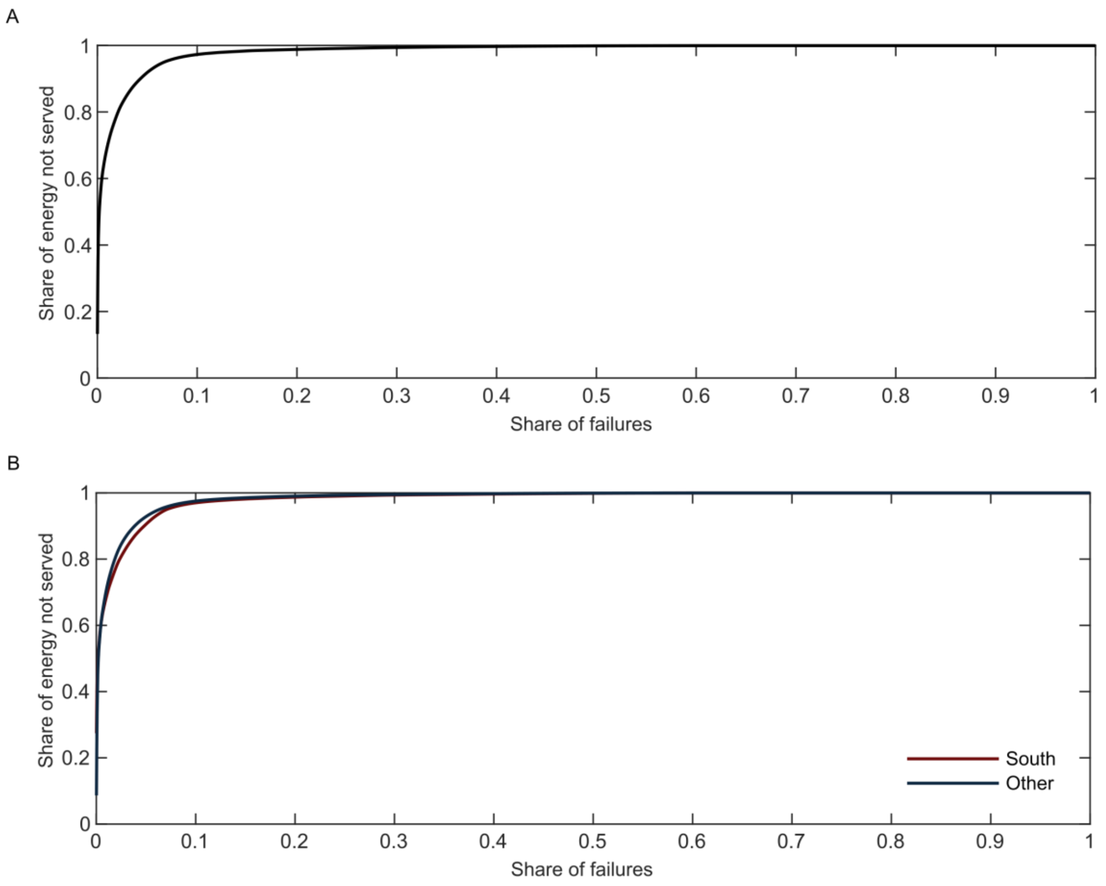

Figure S5. Cumulative distribution function of energy not served, related to Figures 1 and 3

Cumulative distribution function of energy not served across from failure events (A) across all of Italy and (B) in the South versus other areas. Energy not served measured in MWh.

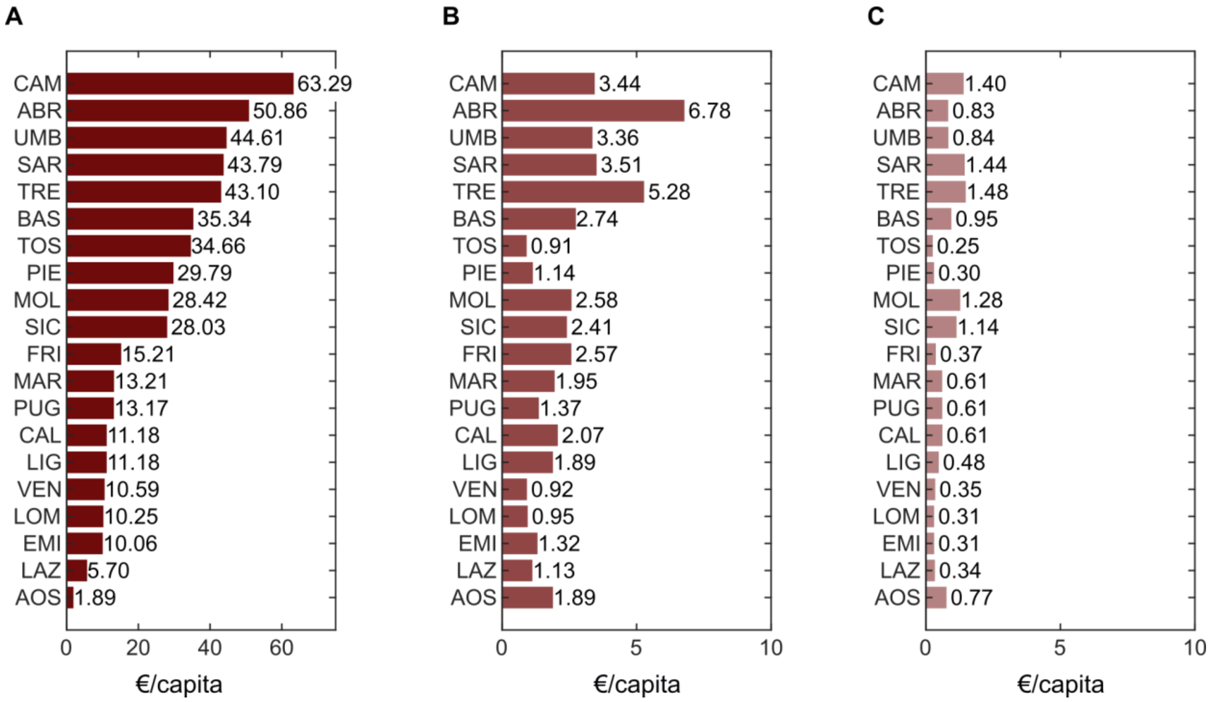

Figure S6. Per capita value of load by region, related to Figure 1

Results shown for (A) 100% of failure events, (B) 95% smallest failure events, and (C) 90% smallest failure events by energy not served (MWh). Regions are unequally affected by large-scale power loss, hence the changing distributions in (A)-(C). Costs shown are totals over the 10-year study period between 2013 and 2022.

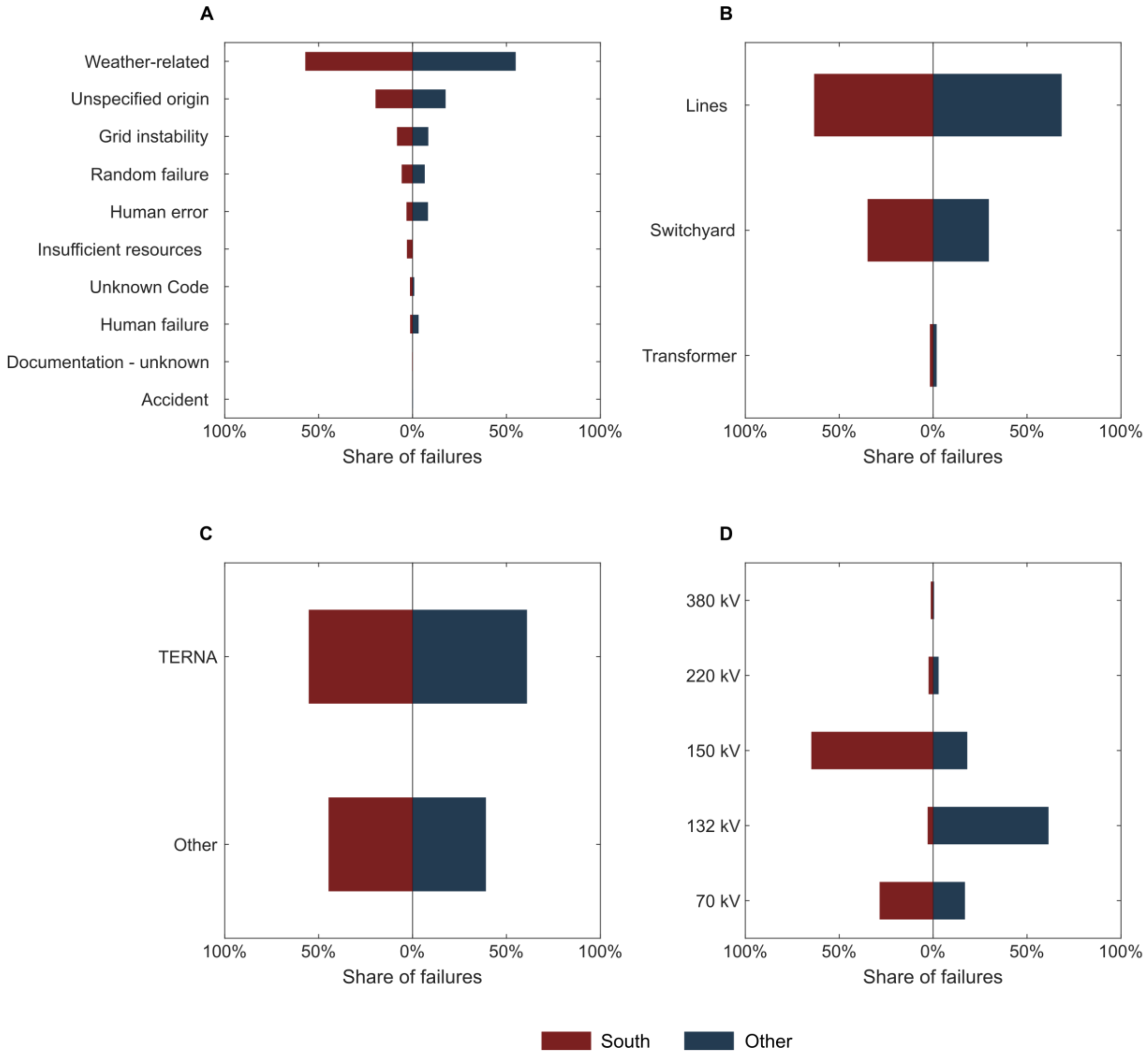

Figure S7. Failure patterns in the South versus other regions, related to Figures 3 and 4

Failure patterns in the South versus other regions according to (A) main cause of failure, (B) affected component, (C) owner of affected component, and (D) voltage level. Note that the distribution of failures per voltage level shown in (D) differs from distribution of all components by voltage level listed in Table S12.

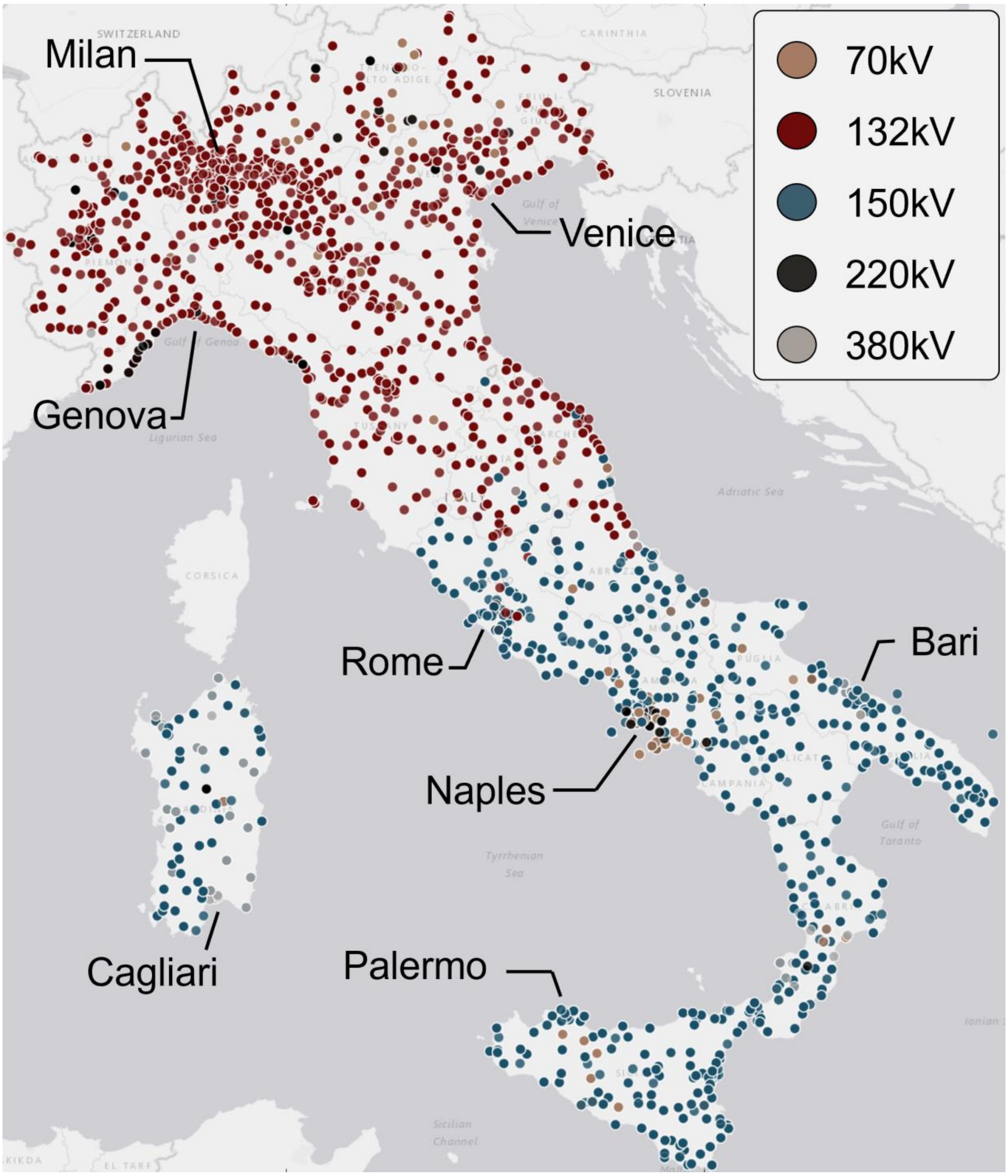

Figure S8. Spatial distribution of substations based on voltage level, related to Figure 3

The figure illustrates the spatial distribution of substations based on the voltage level of failed components. Note the divide between the 132kV voltage, which is more common in the North, and 150kV voltage level, which are more common in the South. The study focuses on the impact to utility consumers, therefore, the number of 220kV and 380kV substations is less common. The high density of substations near the major cities and along the coast is also highlighted.

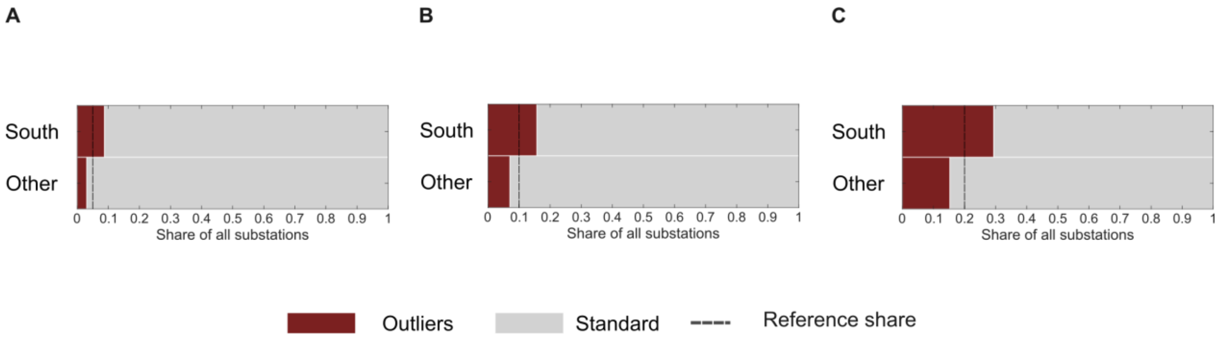

Figure S9. Share of worst-performing substations in the South versus other regions, related to Figure 4

The share of worst-performing substations in the South versus other regions considering the worst (A) 5%, (B) 10%, and (C) 20% of substations according to total number of failures. See Table S14 for results on Fisher's Exact Test.

Table S1. Italian regions and area grouping, related to Figure 1

Full name	Abbreviation	NUTS2 Code	Area grouping
Abruzzo	ABR	ITF1	South
Basilicata	BAS	ITF5	South
Calabria	CAL	ITF6	South
Campania	CAM	ITF3	South
Emilia-Romagna	EMR	ITH5	Northeast
Friuli-Venezia Giulia	FVG	ITH4	Northeast
Lazio	LAZ	ITI4	Center
Liguria	LIG	ITC3	Northwest
Lombardia	LOM	ITC3	Northwest
Marche	MAR	ITI3	Center
Molise	MOL	ITF2	South
Piemonte	PIE	ITC1	Northwest
Puglia	PUG	ITF4	South
Sardegna	SAR	ITG2	Sardegna
Sicilia	SIC	ITG1	South
Toscana	TOS	ITI1	Center
Trentino-Alto Adige	TAA	ITH1/ITH2	Northeast
Umbria	UMB	ITI2	Center
Veneto	VEN	ITH3	Northeast
Valle d'Aosta/Vallée d'Aoste	AOS	ITC2	Northwest

A description of the regional groupings is provided in Geographic scale and scope in Method details.

Table S2. Key social metrics for Italian regions, related to Figure 1

Region	Population (thousands)	CVI	IVI	FI	HDI
Abruzzo	1273	0.53	0.39	0.63	0.89
Basilicata	538	0.66	0.71	0.74	0.86
Calabria	1847	0.72	0.60	0.88	0.85
Campania	5610	0.51	0.40	0.78	0.85
Emilia-Romagna	4438	0.31	0.24	0.37	0.92
Friuli-Venezia Giulia	1194	0.35	0.34	0.39	0.90
Lazio	5721	0.32	0.18	0.66	0.91
Liguria	1508	0.46	0.36	0.52	0.90
Lombardia	9977	0.23	0.19	0.39	0.91
Marche	1484	0.39	0.33	0.39	0.90
Molise	291	0.6	0.73	0.76	0.87
Piemonte	4251	0.38	0.35	0.47	0.90
Puglia	3908	0.54	0.28	0.74	0.85
Sardegna	1578	0.71	0.62	0.77	0.87
Sicilia	4814	0.67	0.48	0.87	0.85
Toscana	3662	0.39	0.35	0.45	0.91
Trentino-Alto Adige	1077	0.14	0.55	0.22	0.92
Umbria	856	0.38	0.37	0.48	0.90
Valle d'Aosta/ Vallée d'Aoste	123	0.31	0.62	0.69	0.89
Veneto	4850	0.26	0.26	0.26	0.90

CVI: Community Vulnerability Index. IVI: infrastructure vulnerability index. FI: Mean regional fragility index developed by the Italian National Institute of Statistics[2]. HDI: Human Development Index[3].

Table S3. Regions and power system performance, related to Figure 1

Region	Energy not served	Failures	Failures per 10k people	Days between failures	Value of lost load per person per year [€ /year]
Abruzzo	3973	508	3.99	6	5.09 (1.88-11.77)
Basilicata	1166	154	2.86	21	3.53 (1.31-8.17)
Calabria	1213	638	3.45	5	1.07 (0.40-2.48)
Campania	21818	1828	3.26	2	6.34 (2.34-14.66)
Emilia-Romagna	2147	399	0.90	8	0.79 (0.29-1.82)
Friuli-Venezia Giulia	1115	258	2.16	13	1.52 (0.56-3.52)
Lazio	2548	690	1.21	5	0.73 (0.27-1.68)
Liguria	1141	371	2.46	9	1.23 (0.46-2.85)
Lombardia	6278	1228	1.23	3	1.03 (0.38-2.37)
Marche	1204	258	1.74	13	1.32 (0.49-3.06)
Molise	507	135	4.64	24	2.84 (1.05-6.58)
Piemonte	7809	663	1.56	5	2.99 (1.11-6.92)
Puglia	3160	788	2.02	4	1.32 (0.49-3.05)
Sardegna	4243	559	3.54	6	4.38 (1.62-10.14)
Sicilia	8287	1613	3.35	2	2.80 (1.04-6.49)
Toscana	7791	348	0.95	9	3.47 (1.28-8.02)
Trentino-Alto Adige	2862	494	4.59	7	4.33 (1.60-10.01)
Umbria	2341	148	1.73	22	4.45 (1.65-10.31)
Veneto	3351	692	1.43	5	1.13 (0.42-2.60)
Valle d’Aosta/Vallée d’Aoste	14	59	4.79	56	0.19 (0.07-0.44)

Energy not served in MWh[5]. Failures: number of all types of failures in 2013-2022[5]. Duration: total duration of all failures in 2013-2022[5]. Population listed in thousands.

Table S4. Estimated values of lost load (VoLL) for the Italian household consumer segment, related to Figure 1

Source	Geographic scope	Estimation method	Estimate [k€/MWh]
Cambridge Economic Policy Associates[6]	Southern Europe	Proxy-based (production function)	6.04 (3.15-11.34)
Marchisio et al.[7]	Italy	Surveys	37.7 (7.5-67.9)
Shivakumar et al.[8]	Italy	Proxy-based (wages)	16.29 (~10-20)

Approximate range given Shivakumar et al[8]. Surveys ask consumers to estimate their own values of lost load, whereas proxy-based methods estimate the value of lost load according to economic indicators and models[9]. Each method has advantages and shortcomings, with consumers not always able to accurately estimate the value of lost power and proxy-based methods not able to account for the time-varying impact of outages (e.g., the impact of an outage in summer versus winter, during the day versus during the night).

Table S5. Predictor variables for regression analysis, related to Figure 2

Predictor variable	Predictor type	Description	Source	Considered in final regression analysis
CVI	Numeric	Community vulnerability index	Multiple	Yes
IVI	Numeric	Infrastructure vulnerability index	Multiple	Yes
i10fg	Numeric	Instantaneous 10-meter wind gust	C3S[10]	Yes
ro	Numeric	Run-off	C3S[10]	Yes
t2m	Numeric	Temperature at 2 meters altitude	C3S[10]	Yes
Voltage level	Categorical	Voltage level of the failing component (70 kV, 132 kV, 150 kV, 220 kV, 380 kV)	Italian national blackouts dataset[5]	Yes

Predictor variables used for regression analyses. Variables were selected considering literature[11–13] and confirmed with a Variance Inflation Factor (VIF). VIF are reported with individual regression results (Table S10). See Characterizing community and infrastructure vulnerability in Method details.

Table S6. Comparison of bias correction approaches – results for line failures per capita, related to Figure 2

Lines						
Model input	Log-likelihood	RMSE	R^2	Adjusted R^2	F-statistic	Residuals
Weighted observations	-804.266	2.17	0.505	0.493	40.8	-
Imputed and weighted observations	-1083.3	3.31	0.491	0.479	43.5	Biased
Imputed and weighted observations (imputed values down-weighted)	-1019	2.84	0.48	0.468	41.6	Biased

Summary model comparison considering 95% of data and regression variables listed in Table S5. Observations are weighted by line shares in each TSO region[14]. Imputed values are derived for components at each voltage level and assigned a failure frequency of zero. Down-weighted zero values are weighted at 0.5 strength of the line shares, e.g., if the assumed component share is 0.30, the down-weighted value is 0.15. Significant values indicated: *: <0.05; **<0.01; ***<0.001.

Table S7. Regression results: lines failures/capita, related to Figure 2

Predictor variable	Estimate	Standard error	t-statistic	p-value
(Intercept)	126.081	60.605	2.080	0.038
ln(CVI)	0.236	0.271	0.871	0.385
ln(IVI)	0.768	0.242	3.180	0.002
ln(i10fg)	0.962	0.661	1.455	0.147
ln(ro)	0.141	0.080	1.764	0.079
ln(t2m)	-24.454	10.730	-2.279	0.023
Voltage_132	1.082	0.136	7.972	0.000
Voltage_150	1.146	0.147	7.773	0.000
Voltage_220	-1.548	0.203	-7.632	0.000
Voltage_380	-0.825	0.460	-1.795	0.073

See Table S5 for an explanation of predictor variables. N = 369 Degrees of freedom: 359. RMSE: 2.17. R^2:0.505. Adj. R^2:0.493. F-statistic versus null model: 40.8 (p-value <0.001).

Table S8. Regression results: switchyard failures/capita, related to Figure 2

Predictor variable	Estimate	Standard error	t-statistic	p-value
(Intercept)	-5.194	59.195	-0.088	0.930
ln(CVI)	-0.219	0.251	-0.874	0.383
ln(IVI)	1.274	0.225	5.669	0.000
ln(i10fg)	1.675	0.588	2.847	0.005
ln(ro)	-0.031	0.070	-0.449	0.653
ln(t2m)	-1.882	10.502	-0.179	0.858
Voltage_132	0.795	0.133	5.974	0.000
Voltage_150	1.147	0.139	8.239	0.000
Voltage_220	-0.427	0.142	-2.999	0.003
Voltage_380	0.538	0.356	1.511	0.132

See Table S5 for an explanation of predictor variables. N = 367. Degrees of freedom: 357. RMSE: 1.99. R^2:0.500. Adj. R^2:0.487. F-statistic versus null model: 39.6 (p-value<0.001).

Table S9. Regression results: transformer failures/capita, related to Figure 2

Predictor variable	Estimate	Standard error	t-statistic	p-value
(Intercept)	22.446	138.580	0.162	0.872
ln(CVI)	-0.164	0.557	-0.294	0.770
ln(IVI)	1.489	0.434	3.434	0.001
ln(i10fg)	0.188	0.937	0.200	0.842
ln(ro)	0.027	0.097	0.278	0.782
ln(t2m)	-6.320	24.604	-0.257	0.798
Voltage_132	0.189	0.229	0.826	0.411
Voltage_150	0.276	0.239	1.157	0.250
Voltage_220	-0.162	0.203	-0.796	0.428
Voltage_380	-0.518	0.592	-0.875	0.384

See Table S5 for an explanation of predictor variables. N = 102. Degrees of freedom: 92. RMSE: 1.71. R^2:0.368. Adj. R^2: 0.307. F-statistic versus null model: 5.96 (p-value<0.001).

Table S10. Variance Inflation Factors for all regressions, related to Figure 2

Component	ln(IVI)	ln(CVI)	ln(i10fg)	ln(ro)	ln(t2m)	Voltage
Lines	1.685	2.568	2.173	1.333	1.371	1.335
Switchyard	1.900	2.563	2.007	1.270	1.482	1.383
Transformer	1.965	2.532	2.075	1.405	1.286	1.569

Table S11. Regression results for alternative model specification: lines failures/capita, related to Figure 2

Predictor variable	Estimate	Standard error	t-statistic	p-value
(Intercept)	227.405	52.202	4.356	0.000
ln(CVI)	0.748	0.220	3.396	0.001
ln(i10fg)	1.829	0.610	3.000	0.003
ln(ro)	0.133	0.081	1.644	0.101
ln(t2m)	-42.706	9.180	-4.652	0.000
Voltage_132	1.045	0.137	7.631	0.000
Voltage_150	1.190	0.149	8.011	0.000
Voltage_220	-1.507	0.205	-7.353	0.000
Voltage_380	-0.801	0.465	-1.721	0.086

See Table S5 for an explanation of predictor variables. N = 369 Degrees of freedom: 360. RMSE: 2.20. R^2:0.492. Adj. R^2:0.480. F-statistic versus null model: 45.1 (p-value <0.001). c.f. Table S7 (main model specification).

Table S12. Regression results for alternative model specification: switchyard failures/capita, related to Figure 2

Predictor variable	Estimate	Standard error	t-statistic	p-value
(Intercept)	193.820	49.393	3.924	0.000
ln(CVI)	0.665	0.203	3.269	0.001
ln(i10fg)	3.295	0.535	6.160	0.000
ln(ro)	-0.043	0.073	-0.597	0.551
ln(t2m)	-37.673	8.696	-4.332	0.000
Voltage_132	0.726	0.138	5.277	0.000
Voltage_150	1.214	0.145	8.406	0.000
Voltage_220	-0.403	0.148	-2.723	0.007
Voltage_380	0.539	0.370	1.456	0.146

See Table S5 for an explanation of predictor variables. N = 367. Degrees of freedom: 358. RMSE: 2.07. R^2:0.456. Adj. R^2:0.444. F-statistic versus null model: 37.566 (p-value<0.001). c.f. Table S8 (main model specification).

Table S13. Regression results for alternative model specification: transformer failures/capita, related to Figure 2

Predictor variable	Estimate	Standard error	t-statistic	p-value
(Intercept)	375.8131	98.043	3.833	0.000
ln(CVI)	1.264	0.391	3.232	0.002
ln(i10fg)	2.006	0.817	2.456	0.016
ln(ro)	-0.008	0.102	-0.075	0.940
ln(t2m)	-69.427	17.280	-4.018	0.000
Voltage_132	0.040	0.238	0.167	0.868
Voltage_150	0.248	0.252	0.982	0.329
Voltage_220	-0.230	0.214	-1.077	0.284
Voltage_380	-0.871	0.616	-1.414	0.161

See Table S5 for an explanation of predictor variables. N = 102. Degrees of freedom: 93. RMSE: 1.81. R^2:0.288. Adj. R^2: 0.226. F-statistic versus null model: 4.692 (p-value<0.001). c.f. Table S9 (main model specification).

Table S14. Results for Fisher's Exact Test and share of worst-performing substations in the South, related to Figure S9

Share of substations considered as "worst"	Group 1	Group 2	Odds ratio	p-value	Confidence interval	Result
5%	South	All others	2.845	<0.001	[1.818, 4.464]	Reject H_0
10%	South	All others	2.230	<0.001	[1.617, 3.075]	Reject H_0
20%	South	All others	1.918	<0.001	[1.512, 2.433]	Reject H_0

H_0: No non-random association between substation location and inclusion in the list of worst-performing substations across Italy by frequency of failure. Rejecting the hypothesis implies that there is a relationship between substation location and inclusion in the list of worst-performing substations.

Table S15. Share of components per voltage level by Italian region, related to Figure 3

Region	70 kV	132 kV	150 kV	220 kV	380 kV
Abruzzo	24%	24%	24%	7%	21%
Basilicata	24%	24%	24%	7%	21%
Calabria	24%	24%	24%	7%	21%
Campania	24%	24%	24%	7%	21%
Emilia-Romagna	24%	24%	24%	15%	14%
Friuli-Venezia Giulia	24%	24%	24%	15%	14%
Lazio	24%	24%	24%	7%	21%
Liguria	21%	21%	21%	22%	16%
Lombardia	21%	21%	21%	22%	16%
Marche	24%	24%	24%	7%	21%
Molise	24%	24%	24%	7%	21%
Piemonte	21%	21%	21%	22%	16%
Puglia	24%	24%	24%	7%	21%
Sardegna	15%	15%	15%	29%	26%
Sicilia	22%	22%	22%	28%	7%
Toscana	24%	24%	24%	15%	14%
Trentino-Alto Adige	24%	24%	24%	15%	14%
Umbria	24%	24%	24%	7%	21%
Veneto	24%	24%	24%	15%	14%
Valle d’Aosta/Vallée d’Aoste	21%	21%	21%	22%	16%

Regional shares based on aggregated values listed for TSO regions[14]. Share of components at 70 kV, 132 kV, and 150 kV assumed to be equal in absence of more precise information.

References

1. OECD, European Union, and Joint Research Centre - European Commission (2008). Handbook on Constructing Composite Indicators: Methodology and User Guide (OECD) https://doi.org/10.1787/9789264043466-en.

2. Istituto Nazionale di Statistica (2023). Municipal Fragility Index. Istat. https://www.istat.it/en/press-release/municipal-fragility-index-ifc/.

3. Global Data Lab (2024). Subnational HDI. Version v8.1. https://globaldatalab.org/shdi/table/shdi/ITA/?levels=1+4&years=2022&interpolation=0&extrapolation=0 https://globaldatalab.org/shdi/table/shdi/ITA/?levels=1+4&years=2022&interpolation=0&extrapolation=0.

4. United Nations Development Programme (2024). Human Development Index (HDI). Human Development Reports. https://hdr.undp.org/data-center/human-development-index#/indicies/HDI.

5. Stankovski, A., Gjorgiev, B., Locher, L., and Sansavini, G. (2023). Power blackouts in Europe: Analyses, key insights, and recommendations from empirical evidence. Joule, S2542435123003665. https://doi.org/10.1016/j.joule.2023.09.005.

6. Cambridge Economic Policy Associates Ltd. (2018). Study on the estimation of the Value of Load Load of electricity supply in Europe (Agency for the Cooperation of Energy Regulators).

7. Marchisio, L., Genoese, F., Vedovelli, F., Salterini, F., and Costa, S. (2022). Estimating the Value of Lost Load in Italy through outage cost surveys. In 2022 AEIT International Annual Conference (AEIT) (IEEE), pp. 1–5. https://doi.org/10.23919/AEIT56783.2022.9951827.

8. Shivakumar, A., Welsch, M., Taliotis, C., Jakšić, D., Baričević, T., Howells, M., Gupta, S., and Rogner, H. (2017). Valuing blackouts and lost leisure: Estimating electricity interruption costs for households across the European Union. Energy Research & Social Science *34*, 39–48. https://doi.org/10.1016/j.erss.2017.05.010.

9. Gorman, W. (2022). The quest to quantify the value of lost load: A critical review of the economics of power outages. The Electricity Journal *35*, 107187. https://doi.org/10.1016/j.tej.2022.107187.

10. C3S (2018). ERA5 hourly data on single levels from 1940 to present. (Copernicus Climate Change Service (C3S) Climate Data Store (CDS)). https://doi.org/10.24381/CDS.ADBB2D47 https://doi.org/10.24381/CDS.ADBB2D47.

11. Sperstad, I.B., Kjølle, G.H., and Gjerde, O. (2020). A comprehensive framework for vulnerability analysis of extraordinary events in power systems. Reliability Engineering & System Safety *196*, 106788. https://doi.org/10.1016/j.ress.2019.106788.

12. International Energy Agency (2021). Climate resilience (International Energy Agency).

13. Verschuur, J., Fernández-Pérez, A., Mühlhofer, E., Nirandjan, S., Borgomeo, E., Becher, O., Voskaki, A., Oughton, E.J., Stankovski, A., Greco, S.F., et al. (2024). Quantifying climate risks to infrastructure systems: A comparative review of developments across infrastructure sectors. PLOS Clim *3*, e0000331. https://doi.org/10.1371/journal.pclm.0000331.

14. CIGRE (2020). Italian Power System (CIGRE).
